# Uterine transplantation and IVF for congenital or acquired uterine factor infertility: A systematic review of safety and efficacy outcomes in the first 52 recipients

**DOI:** 10.1371/journal.pone.0232323

**Published:** 2020-04-29

**Authors:** Jessica Daolio, Stefano Palomba, Simone Paganelli, Angela Falbo, Lorenzo Aguzzoli

**Affiliations:** 1 Department of Obstetrics & Gynaecology, Azienda Unità Sanitaria Locale—IRCCS di Reggio Emilia, Reggio Emilia, Italy; 2 Department of Obstetrics & Gynaecology, Grande Ospedale Metropolitano, Reggio Calabria, Italy; American-Sino Women's and Children's Hospital, CHINA

## Abstract

Uterine transplantation (UTx) associated with IVF restores fertility in women affected by absolute uterine factor infertility (AUFI). Pregnancies achieved both in women undergoing any solid organ transplantation and following IVF are associated with an increased risk of maternal and neonatal complications. This systematic review evaluated this risk in UTx-IVF treated women focusing on the safety and efficacy features of the treatment. Twenty-two studies and three press releases reporting on 52 UTx-IVF treatments were identified. Regarding the safety of treatment, 38/52 (73,1%) of surgical procedures led to the restoration of uterine function in recipients, 12/52 (23,1%) of recipients experienced post-operative complications requiring hysterectomy, and 2/52 (3,8%) of procedures failed before uterine recipients’ surgery due to intra-operative complications. Regarding the efficacy of treatment, results focused on transplanted patients showing full recovery of organ functioning: 16/38 (42,1%) of patients achieved a pregnancy, including two women who gave births twice. UTx-IVF pregnancies led to 16 deliveries and all new-borns were healthy. Six out of 16 (37,5%) UTx pregnancies faced major complications during gestation. Preterm births occurred in 10/16 (62,5%) UTx deliveries. Our data indicates that the risk of gestational and delivery complications deserves important consideration in AUFI women receiving UTx-IVF treatments. However, these observations are preliminary and need to be revised after larger series of data are published.

## Introduction

More than 150,000 women of reproductive age in Europe [1], and approximately 1.5 million women worldwide [2] are affected by absolute uterine factor infertility (AUFI) [3,4]. There are many aetiologies of AUFI, which can be categorized into either congenital or acquired causes that preclude the implantation of an embryo or completion of a pregnancy [5]. AUFI may be due to the anatomical absence of the uterus following hysterectomy or to the Mayer-Rokitansky-Küster-Hauser (MRKH) syndrome [1,6]. Alternatively, AUFI may also manifest as uterine dysfunctions caused by radiotherapy, leiomyoma, the Asherman’s syndrome, or congenital uterine malformations occurred as a consequence of disturbances during the formation, of foetal life or in the development or fusion of the Müllerian ducts [1,6]. Adoption and gestational surrogacy have long been the only two options available for women with AUFI who wish to pursue motherhood, and important legal, ethical and social implications define the feasibility of one option or the other [7,8].

Since 2014, the revolutionary procedure of uterus allotransplantation (UTx) in humans [9,10] has allowed women with AUFI to partially overcome such complications, introducing the possibility of giving birth to a genetically related child [11]. To date, UTx represents another quality-of-life-type improvement in transplantation, and a milestone in the gynaecological field. Furthermore, it has endorsed the concept of reproductive surgery that was previously introduced with the strategy of ovarian tissue transplantation [12]. In fact, both transplantation procedures are performed with the intent of restoring fertility in non-life-saving situations, rather than improving the chance of survival in life-saving situations [13,14].

UTx always requires performing IVF from 6 to 18 months before surgery, due to the conditioning regimen for immunosuppressive therapy, and in order to ascertain fertility between AUFI couples, and finally to reduce the risks concerning bleeding and pelvic infections associated with oocyte retrieval. Specifically, the risk of bleeding is related to the presence of abnormal uterine vascular pedicles and anastomosis sites caused by UTx surgery, and the risk of pelvic infections may constitute a complication of surgical retrieval operations [8,9]. It follows that UTx exposes AUFI women to the risk of complications related to solid organ transplantation (SOT) and/or in vitro fertilization (IVF) treatments, despite the fact that the latter are common and well-documented procedures.

The evaluation of IVF complications is crucial, as the primary endpoint of treatment is the delivery of healthy babies from healthy mothers. From this perspective, the clinical use of the UTx-IVF procedure deserves careful consideration, due to the increased risk of obstetric and neonatal complications related to the IVF procedure itself [15–17]. This is of particular relevance to UTx counselling, as the UTx-IVF procedure is applied with an intention-to-treat care, defined by organ function restoration and the delivery of a healthy offspring.

Despite promising initial achievements [18], the UTx-IVF procedure is still at an early clinical experimental stage, and it faces multiple ongoing challenges. The aim of this systematic review was to provide preliminary data on 1) the UTx-IVF safety related to post-operative, maternal and neonatal complications to which the uterus recipients and the intended children may be potentially exposed to, and on 2) the UTx-IVF efficacy, defined as the number of UTx pregnancies and live births actually available for consultation.

## Materials and methods

This review was conducted in accordance with the Preferred Reporting Items for Systematic Reviews and Meta-Analyses of Individual Participant Data (PRISMA-IPD) guidelines [19]. The review protocol was recorded in PROSPERO [20]; the registry number available for consultation is CRD42016042298 and a copy of the protocol is provided as supporting information (S1 Text). A literature search was performed systematically in order to identify studies involving female patients who underwent UTx to treat AUFI infertility, regardless of whether AUFI was congenital or acquired.

We searched the PubMed, Institute of Biology of the Southern Seas (IBSS), SocINDEX, Institute for Scientific Information, Web of Science, and Google Scholar databases, up to the end of September 2019 by using the following terms alone or in combination: “uterine”, “uterus”, “womb”, “transplant”, “transplantation” and “absolute uterine factor infertility”. A second search was also conducted on common search engines, such as Google, Yahoo, and Mozilla Firefox for the aforementioned terms, in order to identify further cases that were unpublished in scientific reference databases.

All the articles identified by the comprehensive electronic search were examined for the relevance of both title and abstract without any language restriction. Subsequently, articles were screened by full-text availability in English and by content in order to select those eligible for the final analysis. Studies reporting individual data were first considered and used. Additional data on UTx or relevant information from unpublished cases were retrieved by consulting several scientific contributions, such as reviews, original articles and press releases.

In examining the full texts, the following data were extracted to address the primary endpoint of UTx safety: numbers of transplant attempts and the related clinical trial (if registered), country of UTx surgery, donor type, strategy of organ retrieval, graft outcome, graft complications, and the time of graft explantation. To address the secondary outcome of UTx efficacy, the following data were extracted: trial or case number, number of pregnancy attempts by frozen embryo replacement cycles, outcome of the attempt, gestational week at delivery, and obstetric and neonatal complications. The bibliographic search, identification and selection of articles to be included in the final analysis were independently performed by two authors (JD and SPag). Data were independently extrapolated and tabulated by the same authors, and definitively reviewed by a third author (SPal). Any disagreements between operators were assessed, until a consensus among authors was reached.

Initially, the study protocol (PROSPERO ID: CRD42016042298) intended to consider a qualitative and quantitative analysis [19,21]. In addition, a final analysis according to the Oxford Centre for Evidence-based Medicine (OCEM)-Levels of Evidence 2011 guidelines [22] and to the Grading of Recommendations Assessment, Development, and Evaluation (GRADE) system [23] was initially considered.

## Results

**Fig 1:**
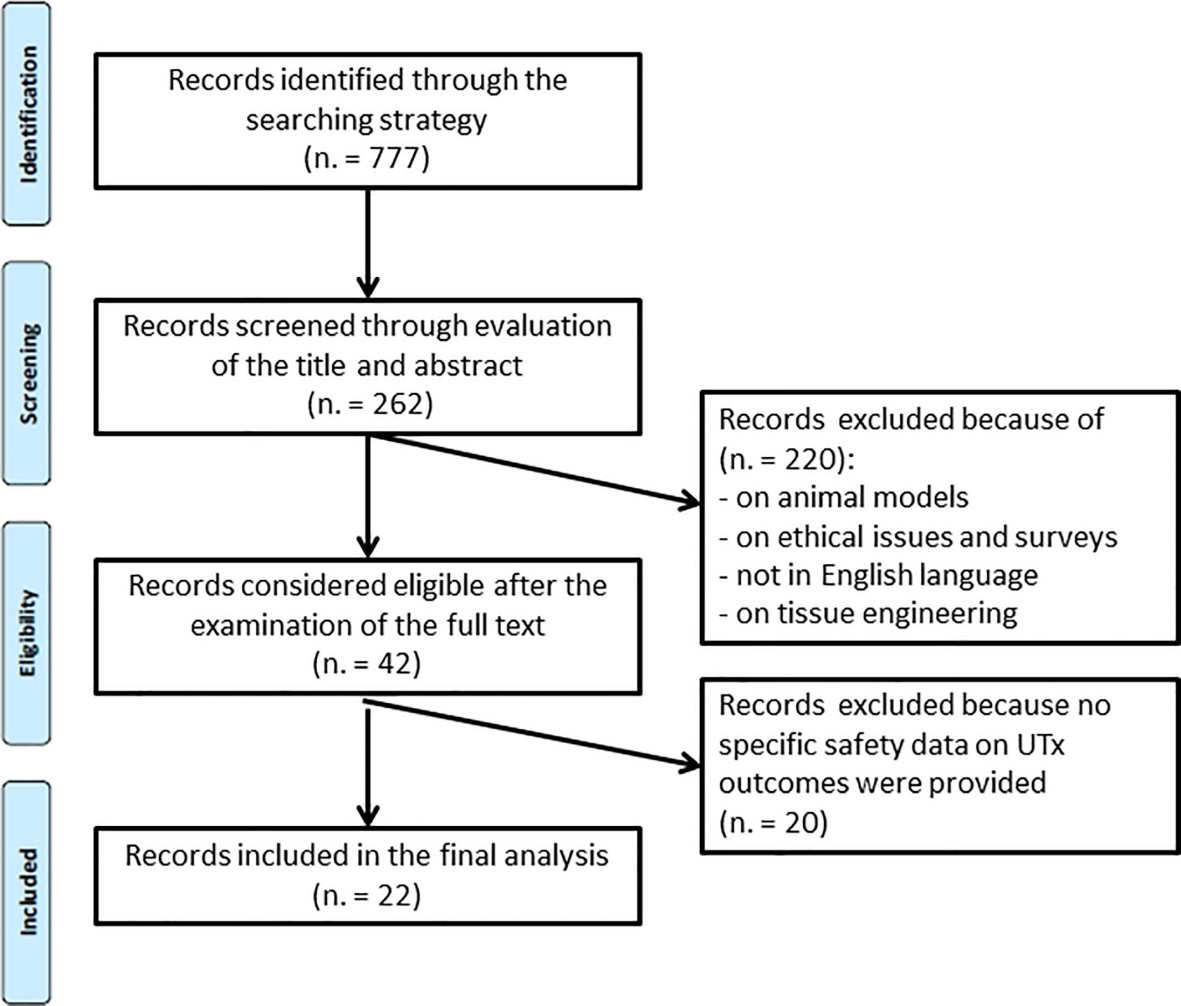
The PRISMA flow diagram illustrates the total number of studies initially considered, included and excluded in this systematic review.

The PRISMA flow diagram (Fig 1) illustrates the total number of studies initially considered, included and excluded in this systematic review. The completed PRISMA-IPD checklist is provided as supporting information (S1 Table).

From a set of 777 studies initially identified through the search strategy, 262 studies were screened throughout the evaluation of title and abstract. After that, 220 studies on animal models, ethical issues, surveys and attitudes, tissue engineering, and in languages other than English were further excluded, including studies providing previously published data without additional and/or new information.

Forty-two studies were then considered eligible for the full-text examination. The final selection included 22 studies: 8 case reports, 6 case series (at least two patients described), 2 articles on the follow-up outcomes of one case report, and 6 reviews/original articles, together with 3 press releases. The final set of studies reporting outcomes on UTx safety and efficacy are cited in Table 1.

**Table 1:** UTx-IVF safety and efficacy outcomes.

Reference (n. of registration)	Transplant n.	Country	N. of patients undergoing UTx	Donor type	Donor surgery	Graft outcome	Hysterectomy	Graft complications leading to hysterectomy	Reference of pregnancy	Pregnancy attempts^§^	Maternal Complications	Live Birth	Gestational week at birth	Neonatal Complications	Note
**Fageeh et al., 2002 [26]**	1	Saudi Arabia	1	LD	LAP	UR	POD99	Thrombosis of bilateral uterine vessels	/						
**Okzan et al., 2013 [27]**	2	Turkey	1	DD	LAP	US	/	None	**Okzan et al., 2016 [28]**	4	NA	0	/	/	/
**Brännström et al., 2014 [9] (Trial n. NCT01844362)**	3	Sweden	9	LD	LAP	UR	POD 105	Persistent intrauterine infection	/						
	4	Sweden		LD	LAP	UR	POD 3	Thrombosis of the uterine arteries	/						
	5	Sweden		LD	LAP	US	/	None	**Brännström et al.,2015 [10]**	1	Preeclampsia	1	31+5	Preterm birth	Maternal URA
	6	Sweden		LD	LAP	US	/	None	**Brännström et al.,2016 [29]**	1	Intrahepatic cholestasis	1	34+4	Preterm birthMild respiratory distress	/
	7	Sweden		LD	LAP	US	/	None	**Castellόn et al., 2017 [30]**	1	Preeclampsia Cholestasis PPROM	1	34+5	Preterm birth	Maternal URA
	8	Sweden		LD	LAP	US	/	None	**Castellόn et al., 2017 [30]**	2*	Preeclampsia	2*	35+3	Preterm birth	Maternal URA *Delivered a 2^nd^ child
	9	Sweden		LD	LAP	US	/	None	**Castellόn et al., 2017 [30]**	2*	NR	2*	35	Preterm birth	*Delivered a 2^nd^ child
	10	Sweden		LD	LAP	US	/	None	**Akouri, 2017 [31]**	1	NR	1	37	Preterm birth	/
	11	Sweden		LD	LAP	US	/	None	**Jones et al., 2019 [32]**	5	NA	0	/	/	/
**Wei et al., 2017 [33] (Trial n. XJZT12Z06)**	12	China	2	LD	RAL	US	/	None	**www.scmp.com/news/china/society/article/2183441/chinas-first-womb-transplant-recipient-gives-birth-healthy-baby [34]**	1	NA	1	34	Preterm birth	NA
	13	China		LD	RAL	US	/	None	**www.scmp.com/news/china/society/article/2183441/chinas-first-womb-transplant-recipient-gives-birth-healthy-baby [34]**	1	NA	NA	NA	NA	NA
**Flyckt et al., 2017 [35] (Trial n.NCT02573415)**	14	Cleveland, US	1	DD	LAP	UR	POD 12	Vascular candidal infection	/						
**Ejzenberg et al., 2019 [36] (National approval n. SNT; 1140/2016)**	15	Brazil	1	DD	LAP	US	/	None	**Ejzenberg et al., 2019 [36]**	1	Pyelonephritis	1	35+3	Preterm birth	/
**Testa et al., 2017 [37] (Trial n.NCT02656550)**	16	Dallas, US	5	LD	LAP	UR	POD 6	Arterial thrombisis	/						
	17	Dallas, US		LD	LAP	UR	POD 12	Graft ischaemia	/						
	18	Dallas, US		LD	LAP	UR	POD 14	Arterial thrombisis	/						
	19	Dallas, US		LD	LAP	US	/	None	**Testa et al.,2018 [38]**	1	Sub-chorionic haematoma	1	33+1	Preterm birth	/
	20	Dallas, US		LD	LAP	US	/	None	**Jones et al., 2019 [32]**	1	None	1	36+6	Preterm birth	/
**Personal Communication in Jones et al., 2019 [32] by Johannesson, 2019**	21	Dallas, US	11	DD	LAP	US	/	None	**Personal Communication in Jones et al., 2019 [32] by Johannesson, 2019**	1	NR	0	NA	/	/
	22	Dallas, US		LD	LAP	US	/	None		1	NR	0	NA	/	/
	23	Dallas, US		DD	LAP	Aborted after donor surgery	Intra-operative	External iliac thrombosis							
	24	Dallas, US		LD	LAP	US	/	None		1	NR	0	NA	/	/
	25	Dallas, US		LD	LAP	UR	POD 1	Graft ischemia							
	26	Dallas, US		LD	LAP	US	/	None		1	NR	0	NA	/	/
	27	Dallas, US		LD	LAP	US	/	None		1	NR	0	NA	/	/
	28	Dallas, US		LD	LAP	US	/	None		1	NR	0	NA	/	
	29	Dallas, US		LD	LAP	UR	POD 3	Post-operative haemoorhage							
	30	Dallas, US		LD	LAP	US	/	None		NR	/	0	NA	/	/
	31	Dallas, US		LD	RAL	US	/	None		NR	/	0	NA	/	/
**Chmel et al., 2018 [39] (Trial n.NCT03277430)**	32	Czech Republic	9	LD	LAP	UR	POD 15	Vascular thrombosis	**Chmel et al., 2018 [39] (Trial n.NCT03277430)**						
	33	Czech Republic		LD	LAP	US	/	None		3	NA	0	NA	NA	/
	34	Czech Republic		LD	LAP	US	/	None		3	NA	0	NA	NA	/
	35	Czech Republic		LD	LAP	US	/	None		NA	/	NA	/	/	/
	36	Czech Republic		LD	LAP	US	/	None		NA	/	NA	/	/	/
	37	Czech Republic		DD	LAP	UR	POM 7	HSV-2 infection							
	38	Czech Republic		DD	LAP	UR	POD 7	Vascular thrombosis							
	39	Czech Republic		DD	LAP	US	/	None		4	NA	0	NA	NA	/
	40	Czech Republic		DD	LAP	US	/	None		2	NA	0	NA	NA	/
**Brucker et al., 2018 [40] (Trial n. NCT03048396)**	41	Germany	3	LD	LAP	Aborted after donor surgery	Intra-operative	Complications of vessels perfusion							
	42	Germany		LD	LAP	US	/	None	**Jones et al., 2019 [32]**	1	NA	1	NA	NA	/
	43	Germany		LD	LAP	US	/	None	**Jones et al., 2019 [32]**	1	NA	0	NA	NA	/
**Brännström et al., 2018 [24]**	44	Serbia	1	LD	LAP	US	/	None	**Brännström et al., 2019 [18]**	1	NA	1	NA	NA	/
**Puntambekar et al., 2018 [41] Puntambekar et al., 2018 [42]**	45	India	4	LD	RAL	US	/	None	**www.dailymail.co.uk/health/article-6393029/Indian-woman-gives-birth-Asias-uterus-transplanted-baby.html [43]**	1	NR	1	NR	NR	/
	46	India		LD	RAL	US	/	None	**Jones et al., 2019 [32]**	1	NR	0	NR	NR	/
	47	India		LD	RAL	US	/	None		NR	/	0	/	/	/
	48	India		LD	RAL	US	/	None		NR	/	0	/	/	/
**Brännström et al., 2018 [44] Brännström, 2018 [25] (Trial n.02987023)**	49	Sweden	4	LD	RAL	US	/	None	**https://nypost.com/2019/04/09/baby-makes-history-afterworlds-first-womb-transplant-performed-by-robot. [45]**	1	NA	1	NA	NA	/
	50	Sweden		LD	RAL	US	/	None		NR	/	0	/	/	/
	51	Sweden		LD	RAL	US	/	None		NR	/	0	/	/	/
	52	Sweden		LD	RAL	US	/	None		NR	/	0	/	/	/
**TOTAL**			52	9 DD 43 LD	41 LAP 11 RAL	2 Aborted 12 UR 38 US				46	6 events	16 births		10 events	

DD = Deceased donor; LAP = Laparotomy; LD = Living donor; NA = not available; NR = Not reported; POD = post operative day; POM = post operative month; RAL = Robotic Assisted Laparoscopy; UR = uterine removal; URA = unilateral maternal agenesis; US = uterine survival.

^§^ each attempt is intended as a frozen embryo replacement cycle followed by a single embryo transfer event

Due to the heterogeneity of the available data, mainly limited to case reports, case series or original articles presenting aggregate data, this systematic review reported only a qualitative analysis, as detailed below.

### UTx safety outcomes

The technical aspects of UTx surgical approaches in donors and recipients have been described in detail elsewhere [18,24,25]. As shown in Table 1, 41/52 (79%) of UTx procurements were performed by laparotomy retrieval surgery and 11/52 (21%) by partial or complete robotic-assisted laparoscopy. In all reported cases, the uterine placement in recipients was performed in an orthotopic position.

Up to the time of writing, 52 UTx surgical procedures had been carried out in different countries (Table 1). Only 34 UTx procedures were from clinical trials for which a registration number was available for consultation. Forty-three out of 52 uterine procurements were from live donors (LDs) and 9/52 were from deceased donors (DDs).

Positive outcomes, id est graft stability and functionality after surgery, were achieved in 33/43 (77%) of LD procedures and in 5/9 (55,5%) of DD procedures, whereas negative outcomes were reported in 10/43 (23,1%) of LD procedures and 4/9 (44%) of DD procedures. Finally, 12/52 (23%) of UTx recipients underwent hysterectomy post-operatively because of vascular complications such as uterine infarction, thrombosis following complications of graft inflow and outflow, or yeast, bacterial or viral infections. Two out of 52 (4%) of UTx procedures aborted before uterine implantation in the recipient, due to unsuccessful organ and vessel perfusion after donor surgery. The incidence of such complications was higher in the DD UTx recipients compared to the LD UTx recipients [4/9 (44%) *vs*. 10/43 (23%), respectively].

### UTx efficacy outcomes

All of UTx recipients underwent at least one IVF cycle, and had their oocytes retrieved and fertilized prior to uterus transplant (Table 2). Different pools of fertilized oocytes and embryos at cleavage or blastocyst stage were frozen, according to the local cryopreservation practice of each IVF Center. To date, at least 46 UTx pregnancy attempts have been performed involving 29 uterine recipients (Table 1): each attempt is intended as a frozen embryo replacement cycle followed by a single embryo transfer (SET) event. SET events were performed 6–18 months after UTx surgery, once assessed the restoration of uterine function in recipients.

**Table 2:** Outcomes of IVF cycles.

Reference (n. of registration)	Number of IVF cycles before UTx surgery	Retrieved oocytes	Number of cryopreserved cells	Cryopreserved cells	Time to first embryo transfer	Note
**Fageeh et al., 2002 [26]**	NR	NR	NR	NR	/	
**Okzan et al., 2013 [27]**	3	At least 15	16	Embryos at cleavage stage	18 months	
**Brännström et al., 2014 [9] (Trial n. NCT01844362)**	2–3	NR	NR	NR	/	
	2–3	NR	NR	NR	/	
	3	18	11	Embryos at cleavage stage	12 months	
	2	15	6	Embryos at blastocyst stage	12 months	
			4	Embryos at cleavage stage		
	2–3	NR	NR	NR	NR	
	2–3	NR	NR	NR	NR	
	2–3	NR	NR	NR	NR	
	2–3	NR	NR	NR	NR	
	2–3	NR	NR	NR	NR	
**Wei et al., 2017 [33] (Trial n. XJZT12Z06)**	2	22	14	NR	12 months	
	NR	NR	NR	NR	NR	
**Flyckt et al., 2017 [35] (Trial n.NCT02573415)**	NR	NR	NR	NR	/	
**Ejzenberg et al., 2019 [36] (National approval n. SNT; 1140/2016)**	1	16	8	Embryos at blastocyst stage	7 months	
**Testa et al., 2017 [37] (Trial n.NCT02656550)**	1	8*	6^	Embryos at blastocyst stage	/	*retrieved embryos ^Euploid (PGS tested)
	2	8*	4^	Embryos at blastocyst stage	/	*retrieved embryos ^Euploid (PGS tested)
	2	7*	4^	Embryos at blastocyst stage	/	*retrieved embryos ^Euploid (PGS tested)
	1	8*	**5^**	Embryos at blastocyst stage	6 months	*retrieved embryos ^Euploid (PGS tested)
	1	7*	**5^**	Embryos at blastocyst stage	NR	*retrieved embryos ^Euploid (PGS tested)
**Personal Communication in Jones et al., 2019 [32] by Johannesson, 2019**	NR	NR	NR	NR	NR	
	NR	NR	NR	NR	NR	
	NR	NR	NR	NR	/	
	NR	NR	NR	NR	NR	
	NR	NR	NR	NR	/	
	NR	NR	NR	NR	NR	
	NR	NR	NR	NR	NR	
	NR	NR	NR	NR	NR	
	NR	NR	NR	NR	/	
	NR	NR	NR	NR	NR	
	NR	NR	NR	NR	NR	
**Chmel et al., 2018 [39] (Trial n.NCT03277430)**	2	23*	10^	Embryos at blastocyst stage	/	* fertilized oocytes ^Euploid
	1	25*	16^	Embryos at blastocyst stage	12–14 months	* fertilized oocytes ^Euploid
	2	34*	12^	Embryos at blastocyst stage	12–14 months	* fertilized oocytes ^Euploid
	3	24*	11^	Embryos at blastocyst stage	NR	* fertilized oocytes ^Euploid
	2	22*	18^	Embryos at blastocyst stage	NR	* fertilized oocytes ^Euploid
	3	22*	10^	Embryos at blastocyst stage	/	* fertilized oocytes ^Euploid
	2	19*	11^	Embryos at blastocyst stage	/	* fertilized oocytes ^Euploid
	1	26*	13^	Embryos at blastocyst stage	12–14 months	* fertilized oocytes ^Euploid
	1	21*	12^	Embryos at blastocyst stage	12–14 months	* fertilized oocytes ^Euploid
**Brucker et al., 2018 [40] (Trial n. NCT03048396)**	2	11	6	fertilized oocytes	/	
	1	18	10	fertilized oocytes	12 months	
	2	9	9	fertilized oocytes	12 months	
**Brännström et al., 2018 [24]**	NR	NR	NR	NR	NR	
**Puntambekar et al., 2018 [41] Puntambekar et al., 2018 [42]**	1	7	4	Embryos at cleavage stage	NR	
	1	11	8	Embryos at cleavage stage	NR	
	NR	NR	NR	NR	NR	
	NR	NR	NR	NR	NR	
**Brännström et al., 2018 [44] Brännström, 2018 [25] (Trial n.02987023)**	NR	NR	NR	NR	NR	
	NR	NR	NR	NR	NR	
	NR	NR	NR	NR	NR	
	NR	NR	NR	NR	NR	

NA = not applicable; NR = Not reported; PGS = Pre-implantation Genetic Screening; URA = unilateral maternal agenesis.

Based on the total number of patients undergoing UTx surgery, 14/52 (27%) of uterine recipients achieved at least one pregnancy. Based on the total number of patients undergoing UTx surgery followed by the restoration of uterine functioning, the ratio switches from 14/52 to 14/38 (37%) of uterine recipients achieving at least one pregnancy.

Considering the total number of patients undergoing a frozen embryo replacement cycle followed by a SET event for which outcomes were available, the UTx pregnancy rate per embryo transfer was 35,5%, corresponding to 16 clinical pregnancies divided by 45 SET.

All pregnant women delivered healthy babies, including two mothers who gave birth twice and accounting for a total of 16/52 (30,8%) and 16/38 (42,1%) of UTx pregnancies and babies. Maternal complications occurred in 6/16 (37,5%) of UTx-IVF pregnancies. Pre-eclampsia was the most faced gestational complication, as it was reported in 3/16 (19%) of UTx-IVF pregnancies. All deliveries were carried out by elective caesarean sections between gestational weeks 31 and 37. Accordingly, preterm birth was reported in 10/16 (62,5%) deliveries, constituting the most recurrent neonatal complication.

Recovery requiring intensive care services was not reported in either UTx mothers or babies.

## Discussion

In the last decade, the UTx-IVF procedure has successfully resulted in several live births, and surgical techniques have been improved for organs of both deceased and living donors. However, the UTx procedure still carries multiple challenges even in the most expert hands. In this systematic review, we collected data on 52 UTx-IVF procedures and 16 live births from an exhaustive bibliographic search conducted by consulting scientific sources and browsing the most common search engines.

The number of studies on UTx is constantly growing, but we identified only a limited set of 22 studies providing data on UTx-IVF cases. By the time of writing, these studies allowed us to objectively make the following estimations: the safety of the UTx-IVF procedure, defined as the number of functioning grafts divided by the number of treated patients, is 73,1% (38/52); the efficacy of the UTx-IVF procedure, defined as the number of live births divided by the number of successfully treated patients, is 42,1% (16/38). Despite the fact that our results were calculated on a small number of cases, they warrant careful consideration prior to expanding the UTx-IVF procedure to new groups of patients in light of the following limitations. Published studies represent only a small percentage of the cases actually performed by the time of writing, and this could influence the accuracy of the estimations of safety and efficacy in both UTx recipients and children, which could be potentially underestimated. As a matter of fact, more than 60 UTx procedures have been performed worldwide, and 18 babies have been delivered, but half of these cases has not yet been scientifically published [32].

The majority of studies does not describe post-operative, maternal and neonatal outcomes simultaneously, probably due to the different timings of occurrence concerning UTx surgery, gestational period and delivery. Data are often published at distance and reported in more than one study, or referred to as personal communications. Furthermore, many studies do not capture the learning curve of the surgical teams at different centers worldwide, and it is to be hoped that further investigations will provide more standardized UTx surgical procedures.

Although the accuracy of UTx-IVF safety and efficacy outcomes cannot be evaluated definitively until UTx centers provide additional, meaningful data from larger series of UTx cases treated homogeneously, we critically evaluate the following aspects.

UTx surgery in recipients is mainly complicated by vascular impairments and uterine infections with a negative impact on graft survival and maintenance [9, 26, 35, 37, 39, 40]. The incidence of post-operative complications leading to hysterectomy in UTx recipients was estimated to be 23%, but this value could be underestimated.

The cohort of UTx patients treated and reported thus far represents only 30% of the entire cohort of UTx patients planned to be enrolled by registered UTx clinical trials (Table 3), together with all individual cases treated following local institutional approvals (Table 4).

**Table 3:** Registered UTx clinical trials.

Identifier number*	Institution, State	Enrolled/Estimated enrollment	Donors	Study completion	Outcome measures	Study phase
**NCT01844362**	Sahlgrenska University Hospital Gothenburg, Sweden	10	LD	April, 2018	TS, PR, LBR	Active, not recruiting
**NCT03138226**	Sahlgrenska University Hospital Gothenburg, Sweden	6	LD	December 2025	LBR	Recruiting
**NCT03277430**	Institute for Clinical and Experimental Medicine Prague, Czech Republic	20	LD/DD	December 2025	TS, LBR LD LBR vs. DD LBR, TC, PC, LD surgery vs. DD surgery	Recruiting
**NCT02656550**	Baylor University Medical Center, Dallas Texas, US	10	LD/DD	January 2026	LBR	Recruiting
**NCT03307356**	University of Pennsylvania, Philadelphia Pennsylvania, US	5	DD	July 2029	TS, TC	Recruiting
**NCT02987023**	Sahlgrenska University Hospital Gothenburg, Sweden	10	LD	December 2022	LBR, newborns follow-up	Recruiting
**NCT02573415**	Cleveland Clinic, Cleveland Florida, US	10	DD	December 2021	LBR, PC, NC	Recruiting
**NCT02637674**	Limoges Hospital Limoges, France	10	DD	January 2022	TS, SMC, TC, PR, PC, LBR	Recruiting
**NCT03252795**	Ghent University Hospital—Women's Clinic Ghent, Belgium	20	DD	December 2023	TS, TC, PR, LBR	Recruiting
**NCT 03590405**	Sahlgrenska University Hospital Gothenburg, Sweden	5	LD	December 2021	LB	Recruiting
**NCT03689842**	Hopital Foch	10	LD	June 1, 2025	PR	Recruiting
**NCT03048396**	University Women's Hospital, Tübingen, Germany	10	LD	December 2019	Number of patients interested in UTx, with a potential donor and having a UTx medical indication	Recruiting by invitation
**NCT03581019**	Sahlgrenska University Hospital Gothenburg, Sweden	8	DD	December 2025	LB of an healthy child	Recruiting by invitation
**NCT02741102**	Brigham and Women's Hospital, Boston Massachusetts, US	10	LD	January 2023	LBR, TC, PC, donor QOL, CC, QOL	Not yet recruiting
**NCT03284073**	Mansoura Urology and Nephrology Center, Mansoura Al-Dakahliya, Egypt	11	LD	October 2019	TS, SMC, PR, LBR	Not yet recruiting
**NCT02388802**	Imperial College London UK	10	DD	January 2020	TS, PR, LBR	Not yet recruiting
**NCT02409147**	University of Nebraska Medical Center, Omaha Nebraska, US	5	DD	January 2025	TS	Suspended due to lack of funding

CC = cost comparison between UTx vs. surrogacy and adoption; DD = deceased donors; LBR = live birth rate; LD = living donors; NC = neonatal complication; PC = pregnancy complications; PR = pregnancy rate; QOL = quality of life; SMC = spontaneous menstruation commencement; TC = transplant complications; TS = transplant success within 12 month post-operatively; UK = United Kingdom; US = United States; UTx = uterus transplantation.

*all trials were recorded on www.ClinicalTrials.gov

**Table 4:** UTx case studies with Institutional approval.

Source	Institution, State	N° of treated cases	Donors
**Fageeh et al., 2002 [26]**	King Fahad Hospital and Research Center, Jeddah, Saudi Arabia	1	LD
**Okzan et al., 2013 [27]**	Akdeniz University, Antalya, Turkey	1	DD
**Ejzenberg et al., 2019 [36]**	Hospital das Clínicas da Faculdade de Medicina da Universidade de São Paulo, São Paulo/SP, Brazil	1	DD
**Wei et al., 2017 [41]**	Xijing Hospital, The Fourth Military Medical University, Xi'an, People's Republic of China	1	LD
**Brännström, 2018 [25]**	Belgrade, Serbia	1	LD
**Puntambekar et al., 2018 [42, 44]**	Galaxy CARE Laparoscopy Institute, Pune, India	4	LD

DD = deceased donors; LD = living donors.

Moreover, there are still unpublished data, and data referred to as personal communications to authors that hinder the ability to perform a high-quality assessment of UTx safety in recipients, which will have to be re-assessed in the future after more UTx procedures will be documented. At the same time, insights into post-operative UTx complications are desirable, in light of the positive attitudes towards this promising infertility treatment. UTx is considered more socially and individually acceptable by women of reproductive age than surrogacy and adoption [46–48], and it is at the forefront of the motherhood options considered by AUFI women [49, 50]. Although several ethical [51–55], psychological [56,57] and social [58–62] issues are actively being debated, overall there is great interest among both LDs and potential recipients towards participating in a UTx trial [63,64].

It is well acknowledged that UTx-IVF efficacy depends on favourable surgical UTx outcome, graft function by 1-year post-operation, and delivery of a healthy baby following a successful frozen embryo replacement cycle. Due to this, IVF deserves consideration as it is important to achieve the main reproductive outcome of the entire procedure. To our best knowledge, this is the first study to collect IVF data from UTx patients in a systematic fashion. The recovery of IVF outcomes was complicated, as it carried multiple challenges. IVF data were available for consultation from very few studies, and IVF treatments were applied according to the local clinical practice of single specialty centers. This highlights different cryopreservation policies that may hamper the standardization of the UTx-IVF procedure by the scientific community.

Nonetheless, the current UTx-IVF efficacy is 42,1% (16/38), based on the number of UTx live births per UTx successful procedures. The UTx pregnancy rate per embryo transfer was 35,5% (16/45). This last might be higher than presented as many patients have not yet receive embryo transfer. Furthermore, it is important to underline that not all the embryos transferred were genetically screened for euploidy.

Six out of 16 UTx pregnancies faced major complications during the gestational period [8,9, 32, 29, 36, 38], causing late preterm births in the majority of UTx deliveries [9,32,29,36,38]. Although these outcomes do not seem to impact on the safety of UTx mothers and babies, as no mothers or babies were admitted to intensive care units or experienced major medical problems, further conclusions should not be drawn. This is due to incomplete and/or limited data on UTx deliveries available to date, that negatively influence quality assessments of the outcomes and final estimations of the risk of maternal and neonatal complications in patients undergoing SOT [65,66]. In fact, the risk of complications in SOT pregnancies, such as pre-eclampsia, foetal growth restriction and preterm birth, is increased compared to the risk associated to spontaneous non-transplanted pregnancies [65,66]. Similar data are reported on pregnant women undergoing IVF in comparison to spontaneous conceptions [15–17,67], prompting us to consider UTx-IVF pregnancies at high risk for complications. Given that, careful multidisciplinary counselling is recommended to help patients cope with the reproductive and gestational aspects [68–70].

Specifically, the incidence of pre-eclampsia in healthy women ranges from 3% to 5%, but in SOT patients it accounts for 21.9% liver and 27% renal transplantations respectively [65]. Similarly, the incidence of pre-eclampsia in UTx pregnancies was of 21%. A plausible explanation is that UTx mothers experience the synergistic effect of several risk factors related to this complication, such as the immunosuppression regimen, the presence of a renal disease and the IVF treatment. However, only further analyses from a larger UTx cohort can provide insights to help clarify this issue. In addition, it has been long believed that the development of pre-eclampsia arises from defective placentation following utero-placental hypoperfusion and hypoxia [71,72]. Since no study has so far investigated this relationship in UTx-IVF placentas specifically, major advances regarding the physiology of UTx-placentation could play a pivotal role in the understanding of the occurrence of gestational complications post-UTx surgery, such as pre-eclampsia.

The condition of late preterm birth, that characterizes all UTx babies delivered thus far, is defined by the World Health Organization as the delivery of babies born between the completion of 32 to 37 weeks of gestation. This condition is associated with a lower risk of major medical consequences compared to very preterm births, defined by the World Health Organization as babies born between the completion of 28 to 32 weeks of gestation [73,74]. Accordingly, none of the UTx new-borns were admitted to neonatal intensive care units, and all of them have been reported as healthy babies to date. The same conclusion was reached by Jones et al. (2019). However, a close short- and long-term follow-up is recommended, as late preterm infants are at an increased risk of adverse neonatal outcomes, long-term neurodevelopmental and behavioural *sequelae*, lower cognitive functioning, and ongoing respiratory morbidities [73].

## Conclusions

Taken together, the results of surgical, obstetric and neonatal outcomes highlight that the application of human UTx, as an infertility treatment that aims to solve AUFI, exposes mothers and babies to the risk of complications during the gestational period and at delivery. Our results are in accordance with those recently provided by Jones et al. (2019), and advocate the use of the international registry created within the International Society of Uterus Transplantation. This tool was created with the aim of collecting negative and positive UTx outcomes, so that important advancements in the field of UTx can be made. This approach will contribute to improving research in the field of surgical refinements, uterine bioengineering, donor selection and screening, organ preservation modalities, immunosuppression schedules, patient safety, and short- and long-term follow-ups. An improvement of the reporting system regarding the number of UTx-IVF attempts is desirable to achieve a standardized UTx procedure, increase the transparency of benefits and risks regarding this innovative infertility treatment, and provide better care, encompassing all of the medical, institutional and social parties involved.

## Supporting information

S1 Text Copy of the protocol. (PDF)

S1 Table PRISMA-IPD checklist. (PDF)
