# Supplementary material for: Uterine transplantation and IVF for congenital or acquired uterine factor infertility: A systematic review of safety and efficacy outcomes in the first 52 recipients
Source: PLoS One. 2020 Apr 29;15(4):e0232323. doi: 10.1371/journal.pone.0232323 (PMC7190173; doi:10.1371/journal.pone.0232323)
Supplement: S1 Text — (PDF) [file pone.0232323.s001.pdf]

PROSPERO International prospective register of systematic reviews

Pregnancy and neonatal outcomes in ovarian and uterine transplant recipients: a systematic review of the literature

Stefano Palomba, Giovanni Battista La Sala, Jessica Daolio

Citation

Stefano Palomba, Giovanni Battista La Sala, Jessica Daolio. Pregnancy and neonatal outcomes in ovarian and uterine transplant recipients: a systematic review of the literature. PROSPERO 2016:CRD42016042298 Available from http://www.crd.york.ac.uk/PROSPERO_REBRANDING/display_record.asp?ID=CRD42016042298

Review question(s)

Is the risk of pregnancy and neonatal complications increased in subfertile patients who received ovarian and uterine transplantation?

Searches

The methodology used for the current review will consist in searching all available articles until August 2016 detailing the maternal and neonatal outcomes in women who received transplant surgery for fertility enhancement (ovary, ovarian tissue, and uterine transplantation). Multiple strategies will be used to search and identify relevant demographic, epidemiological, clinical and experimental studies.

Sociological online libraries (IBSS, SocINDEX), Institute for Scientific Information, Web of Science and Google Scholar, Ovid MEDLINE, EMBASE, Ovid OLDMEDLINE, Pre-MEDLINE, in-Process & Other Non-Indexed NLM, HaPI, and the Cochrane Library will be used as sources. A combination of Medical Subject Headings and words will be employed using ‘AND’ or OR as appropriate. The search will be conducted independently by all Authors.

No restriction for study design or language will be considered.

Types of study to be included

No restrictions on the types of study design will be planned.

Only studies that did not report data on pregnancy and neonatal outcomes will be excluded from analysis.

Condition or domain being studied

The primary endpoint in reproductive medicine should be the healthy baby-in-arms, and all other (clinical and/or biological) endpoints should be considered as surrogates. Nonetheless, most publications of infertility clinical trials do not report clear data about the harms of medical, surgical, and biological procedures for enhancing fertility. In fact, only 4.8% and 5.7% of randomized controlled trials (RCTs) on infertility treatments reported on neonatal and maternal outcomes, respectively. In part, this is due to difficulty in obtaining data since obstetric and neonatal care are delivered by different providers, and patients are lost to follow-up. During the last years a great emphasis has been given to the risk of adverse pregnancy outcomes after assisted reproductive technology (ART), including in vitro fertilization

(IVF) and intracytoplasmic sperm injection (ICSI), when compared with natural conceptions. Obstetric and neonatal risk associated with fertility treatment are influenced by three main determinants: multiple gestation, patients' and/or couples’ characteristics and co-morbidities, and infertility treatments and biological manipulations. Infertility itself is a risk factor for obstetric complications, creating an inherent bias in studies of fertility treatment. More and more pregnancies have been obtained after ovary and/or ovarian tissue transplantation. Similarly, recent findings seem to suggest efficacy in the future of the uterine transplanation for uterine factor of infertility. At the moment no systematic data are available regarding the safety of these surgical procedures in terms of maternal and

neonatal/perinatal health.

Participants/ population

Infertile women who received a surgical procedure of ovarian and/or ovarian tissue or uterine transplantation and had a clinical pregnancy will be included in the analysis. Data on biochemical pregnancies will be excluded.

Intervention(s), exposure(s)

The interventions will include medical (ovarian stimulation protocol, use of drugs for fertility pretervation, etc.), biological (types of cryopreservation of ovarian tissues and of oocytes/embryos) and surgical (for donors and recipients) procedures aimed to obtain a live birth child after transplantation of the entire ovary/ovarian tissue or of the uterus in a subfertile woman. No exclusion criteria will be used.

Comparator(s)/ control

All potential control patients (pregnancies) for each study will be assessed whenever possible. Ideally, controls should include pregnancies obtained after natural conception in a short time (less than 12 months).

Context

No specific criteria will be used in the current systematic review protocol.

Outcome(s)

Primary outcomes

Maternal mortality and fetal/perinatal/neonatal deaths.

Secondary outcomes

Secondary outcomes included maternal and neonatal/perinatal outcomes. Maternal outcomes will include miscarriage, antepartum/postpartum hemorrhage, pregnancy-induced hypertension, preeclampsia, gestational diabetes, preterm labor, cesarean section, labour dystocia, placenta previa/abruption, and all medical/surgical complications at new onset after the intervention and pregnancy. Fetal outcomes will include neonatal compromise (APGAR), malpresentation, preterm premature rupture of membranes, intrauterine growth restriction, preterm birth, small for gestational age, large for gestational age, admission to neonatal intensive care units, hospital re-admission.

Other relevant outcomes reported in the studies will be noted.

At the moment, it is not possible to hypothesize quantitative analyses for primary and secondary outcomes.

Data extraction, (selection and coding)

Titles and abstracts of studies retrieved using the search strategy will be screened independently by two review authors to identify studies that potentially meet the inclusion criteria outlined above. The same authors will assess independently the full text of these potentially eligible studies. Two review authors will extract data from the included studies independently using a standardised form that will include the following information: first author, journal and year of pubblication, study setting, study population and participant demographics and baseline characteristics, details of the intervention(s) and control conditions, information for assessment of the risk of bias. Missing or contradictory data will be requested from study authors. Any disagreement between them will be resolved through discussion with a third author.

Risk of bias (quality) assessment

For each included study, two independent authors will assess the quality of data checking for missing data, data consistency/discrepancies between and among publications of the researchers team, etc. Disagreements between the review authors over the study quality will be resolved with the involvement of a third author.

Strategy for data synthesis

Available data from the included studies we will detailed in a qualitative analysis (when possible) or in a narrative fashion. A sub-analysis according to each specific type of intervention and to population characteristics will be done.

We anticipate that there will be limited possibility to perform a data synthesis or to give an overall risk in consideration of the studies heterogeneity and of the lack in many studies of control group.

Analysis of subgroups or subsets
The current systematic review will explore the risk of pregnancy and neonatal/perinatal complications according to two main subgroups: patients who received a ovary/ovarian tissue transplantation and patients who had an uterine transplantation. Other sub-analyses will take account of specific indications for the treatment, assisted or natural conceptions, and types of assisted conceptions.

Dissemination plans
Once data have been collected and analyzed the results will be presented at scientific National and International meetings. A manuscript will be prepared and submitted for consideration of peer-reviewed publication.

Contact details for further information
Professor Palomba

Viale Risorgimeto 80 - 42123 Reggio Emilia - Italy

stefanopalomba@tin.it

Organisational affiliation of the review
None

Not available

Review team
Professor Stefano Palomba, ASMN-IRCCS of Reggio Emilia - Italy
Professor Giovanni Battista La Sala, University of Modena and Reggio Emilia - Italy
Dr Jessica Daolio, ASMN-IRCCS of Reggio Emilia - Italy

Collaborators
Ms Valeria Davoli, ASMN-IRCCS of Reggio Emilia - Italy.

Anticipated or actual start date
11 July 2016

Anticipated completion date
30 September 2016

Funding sources/sponsors
Departmental funds from the Center of Reproductive Medicine and Surgery - ASMN-IRCCS of Reggio Emilia (Italy) will be used to support the Authors throughout the study period and manuscript preparation.

Conflicts of interest
None known

Language
English

Country
Italy

Subject index terms status
Subject indexing assigned by CRD

Subject index terms
Female; Humans; Pregnancy; Pregnancy Outcome; Transplant Recipients; Uterus

Stage of review
Ongoing

Date of registration in PROSPERO
04 July 2016

Date of publication of this revision
04 July 2016

Stage of review at time of this submission	Started	Completed
Preliminary searches	No	No
Piloting of the study selection process	No	No
Formal screening of search results against eligibility criteria	No	No
Data extraction	No	No
Risk of bias (quality) assessment	No	No
Data analysis	No	No

PROSPERO
International prospective register of systematic reviews
The information in this record has been provided by the named contact for this review. CRD has accepted this information in good faith and registered the review in PROSPERO. CRD bears no responsibility or liability for the content of this registration record, any associated files or external websites.
